# Customizing individual heat mitigation strategies to optimize performance in elite athletes

**DOI:** 10.3389/fphys.2025.1380645

**Published:** 2025-04-16

**Authors:** Yasuki Sekiguchi, William M. Adams, Yuri Hosokawa, Courteney L. Benjamin, Rebecca L. Stearns, Robert A. Huggins, Douglas J. Casa

**Affiliations:** ^1^ Sports Performance Laboratory, Department of Kinesiology and Sport Management, Texas Tech University, Lubbock, TX, United States; ^2^ Department of Sports Medicine, United States Olympic and Paralympic Committee, Colorado Springs, CO, United States; ^3^ United States Coalition for the Prevention of Illness and Injury in Sport, Colorado Springs, CO, United States; ^4^ Department of Kinesiology, University of North Carolina at Greensboro, Greensboro, NC, United States; ^5^ School of Sport, Exercise and Health Sciences, Loughborough University, Leicestershire, United Kingdom; ^6^ Faculty of Sport Sciences, Waseda University, Tokorozawa, Japan; ^7^ Department of Kinesiology, Samford University, Birmingham, AL, United States; ^8^ Korey Stringer Institute, Department of Kinesiology, University of Connecticut, Storrs, CT, United States

**Keywords:** heat acclimation, heat acclimatization, cooling, hydration, athlete performance

## Abstract

The aim of this review is twofold: 1) provide a brief discussion surrounding the interindividual variability that has been observed within the context of heat acclimation/acclimatization, body cooling, and hydration strategies, and 2) provide the reader with a practitioner-focused approach for creating individualized heat mitigation strategies. Considering individual variability for heat acclimation and heat acclimatization, various body cooling strategies, and hydration assessment/fluid replacement is important to maximize effects of these strategies, which lead to better performance and health outcomes. There are many factors to consider, and comprehensive approaches are required. The evidenced-informed decision is critical when making an individual approach, and data will help to make decisions effectively. It is important to keep adjusting the approach based on observed data as data is useful information to check if the approach is effective. Specific considerations to individualize the plan are discussed in this review.

## Introduction

The associated thermoregulatory, (Sawka et al., 1992; Galloway and Maughan, 1997; González-Alonso et al., 1999; Otani et al., 2017), cardiovascular, (González-Alonso et al., 1999; Nassis and Geladas, 2002), metabolic, (Jentjens et al., 2002), and neuromuscular (Nybo and Nielsen, 2001; Morrison et al., 2004; Baillot et al., 2021) changes that occur during exercise in the heat are known to result in declines in aerobic, (Galloway and Maughan, 1997), repeated sprint, (Drust et al., 2005), repeated force production, (Thomas et al., 2006), and cognitive (Racinais et al., 2008) performance. These physiologic (Sawka et al., 1985; Sawka et al., 1992; Mn, 1992; González-Alonso et al., 1995; González-Alonso et al., 1997; Logan-Sprenger et al., 2012; Chou et al., 2018) and performance (Stearns et al., 2009; Casa et al., 2010; Lopez et al., 2011; Distefano et al., 2013; Otani et al., 2016; Adams et al., 2017) outcomes in response to exercise heat stress are further exacerbated by dehydration, which often accompanies exercise. Further, the metabolic heat production from exercise in combination with exposure to environmental heat increases the risk of heat related illness. Given the consequences that can occur from exercising heat stress, it is imperative that effective mitigation strategies are in place to optimize human health and performance.

To mitigate performance-related declines and heat-related illness risk, heat acclimation (HA) (performed in the artificial environmental chamber), heat acclimatization (HAz) (naturally occurring), body cooling, and hydration strategies have been shown to be impactful when used in practical settings. (Marino, 2002; Yeargin et al., 2006; Lorenzo et al., 2010; Chalmers et al., 2014; Alhadad et al., 2019; Douzi et al., 2019; Kerr et al., 2019; Racinais et al., 2021). However, inter-individual differences associated with factors such as fitness level, (Alhadad et al., 2019), anthropometric, (Wickham et al., 2021), biological sex, (Wickham et al., 2021), gene expression, (Sonna et al., 2007), and external factors, such as exercise intensity, (Maunder et al., 2020), volume, (Pryor et al., 2019), and environmental conditions (Galloway and Maughan, 1997) may influence the effectiveness of these strategies. While there is knowledge on the general effects of exercise heat stress on health and performance outcomes, and how individual variability may alter the magnitude of the efficacy of associated heat mitigation strategies, there is no literature on how to develop effective individualized strategies to optimize the former outcomes. Furthermore, traditionally these strategies are performed in isolation with little attention paid to the synergistic effects that each strategy or factor has on the achievement of the goal. Therefore, the aim of this narrative review is twofold: 1) provide a brief discussion surrounding the interindividual variability that has been observed within the context of heat acclimation/acclimatization, body cooling, and hydration strategies, and 2) provide the reader with a practitioner-focused approach for creating individualized heat mitigation strategies. The focus of this paper is professional and elite sports situations where appropriate staff, resources, and money are available to maximize heat mitigation strategies while logistical and environmental constrains still may apply to this setting.

## Heat acclimation and heat acclimatization

Heat acclimation and heat acclimatization are some of the most impactful heat mitigation strategies (Tyler et al., 2016). Both HA and HAz utilize intentional and structured repeated exposures to hot environments that are designed to elicit an increase in internal body temperature and induce profuse sweating; the associated physiological and psychological adaptations occurring as a result of the stress on the body improves physical performance and decreases the risk of exertional heat illness (Armstrong et al., 1987; Pandolf, 1998; Shapiro et al., 1998; Benjamin et al., 2021d; Sekiguchi et al., 2021a). It is important to note that 10–14 days of repeated heat exposure are optimal for inducing most of the physiological adaptions incurred by HA/HAz. Following the induction of HA/HAz, the utilization of intermittent heat training (IHT; e.g., 1–2 d/wk of heat exposure) is an effective method to minimize the extent of decay (Daanen et al., 2018), and extend the period of time an individual can benefit from the physiologic adaptions that have occurred (Benjamin et al., 2021a; Sekiguchi et al., 2021b). Minimizing the magnitude of HA/HAz decay improves the feasibility of using this strategy in and around competition, while also optimizing performance by lowering fatigue and enhancing readiness (Benjamin et al., 2021a; Sekiguchi et al., 2021a).

Many factors have been shown to impact the magnitude of the physiologic adaptations induced by HA/HAz (Périard et al., 2015; Tyler et al., 2016). External factors such as exercise intensity and duration, length and frequency of heat exposure, recovery from heat exposure, and different settings (e.g., different types of sports, occupational, military) (Sekiguchi et al., 2021b), and internal factors, such as fitness level (Alkemade et al., 2021), lean body mass (Wickham et al., 2021), body fat percentage (Wickham et al., 2021), body surface area (Wickham et al., 2021), biologic sex differences (Wickham et al., 2021), gene characteristics (Périard et al., 2015), and body’s response to heat prior to HA/HAz (Ravanelli et al., 2019), collectively influence the body’s response to heat exposure. Additionally, adaptations in different variables (e.g., physiological, thermoregulatory) are independent and an adaptation in one variable does not necessarily demonstrate the similar adaptation to other variables (Corbett et al., 2018). As a result, each individual responds to heat exposure differently, which leads to individual differences in the magnitude of induction, decay, and maintenance of HA/HAz associated adaptations.

**FIGURE 1:**
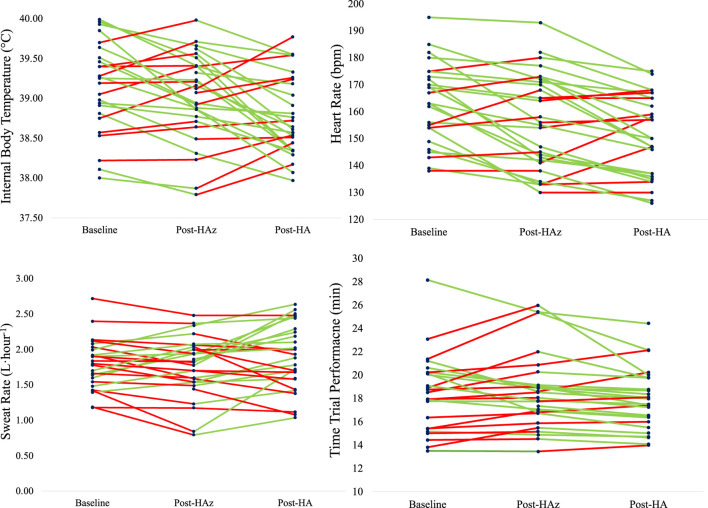
Individual responses in internal body temperature, heart rate, sweat rate, and time trial performance to heat acclimatization (HAz, summer training) and heat acclimation (HA, 5 days of heat exposure in the laboratory) in endurance-trained athletes (Benjamin et al., 2021d; Sekiguchi et al., 2021a). Green line indicates individuals who demonstrated the positive adaptations, and red line indicates who did not show the positive adaptations. Internal body temperature: 60% improved from HAz, 68% improved from HA. Heart rate: 76% improved from HAz, 68% improved from HA. Sweat rate: 36% improved from HAz, 64% improved from HA. Time trial performance: 56% improved from HAz, 78% improved from HA.

Our previous work (Benjamin et al., 2021d; 2021a; Sekiguchi et al., 2021a) explored HA/HAz induction, maintenance and decay among endurance-trained athletes. In this study, participants underwent a period of HAz via outdoor endurance training throughout the summer months. This was then followed by 5 days of HA within a controlled laboratory setting and then 8 weeks of maintenance where participants were assigned either a 1 d/wk IHT exposure, 2 d/wk IHT exposure, or no IHT exposure to determine the changes in associated physiologic and performance adaptations across the testing period. Figure 1 indicates individual responses to HAz and HA from this work (Benjamin et al., 2021d; Sekiguchi et al., 2021b). Individual variability of adaptations in internal body temperature, heart rate, sweat rate, and time trial performance to HAz and HA can be observed, indicated by the color of the line (Green: positive adaptation, Red: not positive adaptation) as well as the slope of the line which demonstrates the magnitude of adaptations (Figure 1). Further, Table 1 demonstrates the range of adaptations with HAz and HA and the degree of maintenance during 8 weeks of IHT between individuals (Benjamin et al., 2021d; Benjamin et al., 20212021a; Sekiguchi et al., 2021a). It can be observed within Figure 1; Table 1 that the magnitude of physiological (e.g., internal body temperature, heart rate, sweating responses) and performance (time trial) adaptations to HA, HAz, and IHT, demonstrated individual differences despite participants adhering to the similar methodologic protocol.

**TABLE 1:** The range of adaptations to heat acclimatization (HAz), heat acclimation (HA), and the degree of maintenance with no intermittent heat training (IHT), once per week of IHT, and twice per week of IHT for 8 weeks (Benjamin et al., 2021a; Sekiguchi et al., 2021b). Data is described as the range of each individual (mean ± standard deviation) of the group in percent change.

%	Core temperature	Heart rate	Sweat rate	Sweat Na concentration	Time trial
HAZ	−1.1 to 2.5 (0.4 ±0.9)	−8.4 to 16.9 (4.7 ± 6.7)	−69.0 to 16.6 (−5.0 ± 19.9)	−75. 6 to 50.4 (−5.1±29.6)	−19.9 to 15.7 (0.2± 8.7)
HA	−1.7 to 3.0 (0.7 ± 1.2)	−12.1 to 13.3 (3.8 ± 6.3)	−29.7 to 104.8 (9.9±25.7)	−17.3 to 76.8 (21.9 ± 25.0)	−8.8 to 22.9 (3.1 ± 6.1)
No-IHT	−4.0 to -0.5 (−1.9±1.5)	−26.7 to 0.6 (−14.3 ± 9.0)	−60.0 to 2.1 (−19.3 ± 19.5)	−163.7 to 11.4 (−68.3 ± 54.7)	−48.3 to 4.0 (−11.5± 16.9)
Once per week IHT	−2.5 to 2.3 (−0.5 ± 1.4)	−17.7 to 4.9 (−5.2 ± 7.5)	−43.4 to 2.9 (−12.2 ± 17.4)	−105.5 to −0.8 (−39.7 ± 38.5)	−6.6 to 8.8 (−1.6 ± 6.2)
Twice per week IHT	−1.5 to 0.9 (−0.2 ± 0.8)	−11.2 to 10.1 (−1.5 ± 6.1)	−191.4 to 35.5 (19.2 ± 62.8)	−108.7 to 35.9 (−49.6 ± 111.6)	−5.0 to 12.9 (3.9 ± 4.9)

Considering the individual differences observed in our work, (Alkemade et al., 2021; Benjamin et al., 2021d; Benjamin et al., 20212021a; Sekiguchi et al., 2021b; Saillant et al., 2022), it is critical to create individualized HAz, HA, and IHT plans. While many other factors can be considered to customize the plan, for example, individuals who do not show gradual adaptions with a few times (3–4 days) of heat exposure as some adaptations should begin to be observed around this time, (Armstrong and Maresh, 1991; Périard et al., 2015), most likely require longer or stronger heat exposure to induce adaptations by increasing the duration, intensity, or environmental conditions. On the other hand, individuals who respond to adaptations with heat exposure well might not need the long length of heat exposure compared to others as evidence indicates 5 days of HA can induce adaptations including sweat rate when the magnitude of heat exposure is high (Benjamin et al., 2021d). This also applies to IHT following HA and/or HAz, and the sports scientist or other responsible personnel should observe the individual response to heat exposure during IHT and decide the number of heat exposures per week to prevent decay (Benjamin et al., 2021a; Sekiguchi et al., 2021a). Additionally, the appropriate amount of recovery from heat exposure might be different for each individual, and it is critical to decide the balance between heat exposure and recovery by monitoring individual response and the level of adaptations (Weller et al., 2007; Benjamin et al., 2021d; Benjamin et al., 2021a; Sekiguchi et al., 2021b). Readiness, recovery, and fitness status can be changed each day after training in the heat based on prescribed training load and the progress of adaptations to heat exposure (Buchheit et al., 2013). It is recommended to adjust training intensity, volume and recovery practices based on individual response in order to induce optimal heat adaptations but not accumulated fatigue or maladaptation (Casadio et al., 2017). For example, training intensity and volume for sports training should decrease when an athlete’s overall workload exceeds the level to which she/he has been exposed or indicated an excessive fatigue level that might not be tolerable.

A consideration to incorporate alongside the implementation of HA/HAz strategies in elite sport is an athlete’s heat tolerance and responsiveness. This is most important for athletes with a recent history of sustaining an exertional heat stroke (EHS) where they may be a risk of heat intolerance. Testing one’s heat tolerance should be integrated into the individualized HA/HAz of the graded rehabilitation following EHS with the intention to assess the individual’s heat tolerance and examine their response to heat exposure. While the known prevalence of heat intolerance is low, this tertiary injury prevention strategy should be regarded as an added component for ensuring a comprehensive approach to HA/HAz.

The intensity and environmental conditions of the testing should be guided by the anticipated levels at which the athlete will experience these respective factors, otherwise the response observed from testing might not provide meaningful information (Ravanelli et al., 2019). Additionally, sport specificity is critical, therefore the frequency, intensity, time, and type of testing need to be considered when deciding testing (Sekiguchi et al., 2021a). For example, it might be important for intermittent athletes to use intermittent exercise for testing to mimic exercise patter along with intensity and duration of exercise while steady-state exercise can be used for endurance athletes. In addition, using objective and reproducible methods to assess heat tolerance and response to heat exposure can aid the practitioner in determining the effectiveness of the rehabilitation program during the implementation of HA/HAz (Sekiguchi et al., 2021b). The protocol should collect all relevant physiologic variables that the practitioner is able to, in addition to all safety related variables (e.g., internal body temperature, heart rate etc.).

## Body cooling

Pre-, per-, and post-body cooling strategies are popular methods used by athletes to optimize athletic recovery and performance when exposed to environmental heat stress (Racinais et al., 2015; Bongers et al., 2017). Meta-analytical reviews on pre-cooling and per-cooling on performance benefits (e.g., finish time, completed distance, time to exhaustion, power output, intermittent sprint, time trial) generally demonstrate improvements, with greater improvements in endurance performance (Jones et al., 2012; Wegmann et al., 2012; Bongers et al., 2015; Jiang et al., 2022). Selection of the ideal cooling strategy will depend on varying sport-specific considerations (e.g., timing, frequency, accessible body part, number of athletes), environmental conditions (e.g., dry-heat, wet-heat, airflow), available resources, the objective for body cooling (e.g., reduction in internal body temperature, improved exercise performance, improved perceived heat stress), and individual preferences (Adams et al., 2016). Outside of the cooling capacity of a given cooling method or modality, the ability to reduce internal body temperature is proportional to the temperature of the cooling source, frequency, duration, and size of the body surface area cooled (Bongers et al., 2017). A mixed cooling method that utilizes multiple cooling strategies is more effective than a single strategy alone (Bongers et al., 2015; 2017), with a cooling strategy that can target the core (e.g., face, neck, head, torso) exhibiting greater performance benefits than those that are applied on limbs only (Jiang et al., 2022). Short duration exercise performance can increase 2%–5% as muscle temperature increases 1°C, but those performance impair as core temperature increases (Racinais and Oksa, 2010). In this case, an ideal cooling method will be maintaining muscle temperatures while keeping core temperature lower. In general, cooling during exercise can enhance both aerobic and anaerobic exercise performance with a greater benefit for aerobic exercise (Douzi et al., 2019). Furthermore, the effect of body cooling on skill performance seems to be case by case as pre-cooling by cold water immersion (CWI) decreased throwing accuracy in American football (Bradley et al., 2019) but per-cooling by ice slurry ingestion did not affect pitching accuracy in softball (Numata et al., 2023). Therefore, the type of sports are important factors to consider when implementing cooling.

A few studies have explored the influence of individual factors that may alter the impact of these cooling strategies. Body fat percentage (Lemire et al., 2009; Godek et al., 2017; Koenig et al., 2022), body mass or lean body mass (Lemire et al., 2009; Godek et al., 2017; Koenig et al., 2022), body surface area (Godek et al., 2017), and body surface area to body mass or lean body mass ratio (Lemire et al., 2009; Godek et al., 2017; Koenig et al., 2022), have all been shown to be important determinants of cooling rates during CWI with hyperthermia. Athletes with a considerably high body fat percentage (approximately 30% or greater) exhibited slow CWI cooling rates when compared to lean (approximately 10%) athletes (Godek et al., 2017). However, the influence of body fat percentage may be minimal when it falls in the range of 10%–30% (Lemire et al., 2009; Koenig et al., 2022). Furthermore, the influence of body surface area, body mass, lean body mass, and body surface area to body mass or lean body mass ratio on cooling rates is inconclusive with mixed results (Lemire et al., 2009; Koenig et al., 2022). It should be noted that these findings are limited to the results from studies that utilized CWI, which has the strongest cooling capacity among the common cooling strategies (Racinais et al., 2015). Future studies are warranted to investigate the impact of morphological characteristics on cooling rates in other external cooling methods that utilize convection and conduction as the primary means of heat transfer to identify and design an individualized body cooling method. Internal body cooling strategies (e.g., ice slurry ingestion, cold fluid ingestion) have also gained popularity among athletes (Racinais et al., 2015). Iwata et al. investigated the influence of sex (male, n = 12, body mass = 65.8 ± 10.3 kg; female, n = 12, body mass = 58.2 ± 10.0 kg) on cooling rates in a study that prescribed 7.5g kg^−1^ ice slurry ingestion during exercise (Iwata et al., 2020). They found no difference in cooling rates, which is likely attributed to the fact that the ingestion amount is adjusted by participants’ body mass. However, further studies will be required to investigate sex differences in body cooling and its effect on performance and recovery (Hutchins et al., 2021).

**FIGURE 2:**
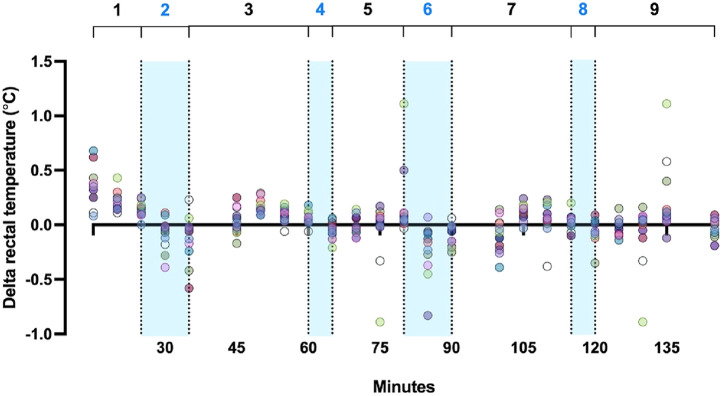
Rectal temperature data extracted from a simulated soccer match study using ice-water dousing by Benjamin et al. (2021b): (1) warm-up + performance battery + intermittent exercise, (2) first 10-min cooling break, (3) performance battery + intermittent exercise, (4) first 5-min cooling break, (5) intermittent exercise, (6) second 10-min cooling break, (7) performance battery + intermittent exercise, (8) second 5-min cooling break, (9) intermittent exercise + performance battery. Note that range of change in rectal temperature during cooling (blue shaded area) varies by individual.

Lastly, the majority of studies that tested the efficacy of body cooling strategies under heat stress were conducted in laboratory settings, with relatively small sample sizes (Jones et al., 2012; Bongers et al., 2015; Rodríguez et al., 2020; Cao et al., 2022; Jiang et al., 2022). This brings a challenge in interpreting and applying results to real-world settings that account for individual variabilities since the study participant recruitment usually selects individuals with similar morphological or physical characteristics to examine the influence of a given cooling strategy (Jones et al., 2012). Therefore, due to the paucity of research in identifying reasons for individual variability associated with various body cooling methods (Figure 2), customization of optimal body cooling strategies should rather focus on the type of sports, timing, and feasibility of implementation, while gauging the maximal cooling capacity of a given method can offer. Also, it is extremely important to consider which part of the body needs to be cooled vs. not to maximize the cooling effects but also not to cause the negative effect of body cooling in some sports.

## Hydration and body fluid balance

Water is the most essential nutrient to sustain life, and in the context of sport, is an important consideration for optimizing health and performance. Despite the advancements in the understanding of body water regulation and the development of evidence-informed strategies to mitigate acute water losses, the dynamic nature of body water homeostasis and associated complexities driving hydration status, gaps in the body of knowledge in the context of sport and personalized hydration strategies persist. In the context of optimizing hydration strategies on an individual level, considerations surrounding the assessment of hydration status and the utilization of individualized fluid intake plans must be prioritized. The following sections provide a succinct overview related to the day-to-day fluctuation of hydration status at the intra- and inter-individual levels followed by considerations needed to inform proper fluid intake strategies.

### Hydration status

Sports coaches, strength and conditioning coaches, and sports medicine professionals commonly instruct their athletes to arrive at the next training session or game ‘hydrated’. While there are good intentions behind this encouragement, the application is often lost in translation. This notion of ‘being hydrated’ is difficult to confidently achieve for athletes and fitness enthusiasts alike because fluid movement is dynamic in the human body and behavioral and socioeconomic factors influence fluid intake behavior, (Armstrong and Kavouras, 2019), and there is not a gold standard way to assess hydration (Armstrong, 2007). Rather, scientists and professionals who work with athletes must build a case for a particular person’s hydration status by completing several valid assessments, including urine color, urine specific gravity (USG), urine and plasma osmolality, plasma volume changes following exercise, thirst sensation, assessment of fluid regulatory hormones such as arginine vasopressin, and body mass changes or succumb to selecting the single most feasible method that may not capture the true hydration state of that athlete. In an athletic setting where establishing hydration status is commonplace, it is critical that those responsible for assessing and communicating hydration status to athletes consider several factors and the potential for error when interpreting hydration data. Therefore, in the field setting, the weight, urine color, and thirst (WUT) Venn diagram is suggested to assess hydration status, especially on the filed settings (Adams et al., 2024; Keefe et al., 2024; Keefe et al., 2025). Assessing hydration status appropriately is a key first step to create hydration strategy to mitigate negative impact of heat by optimizing fluid balance as without understanding of the current hydration status, it is not feasible to creative effective plan.

Body mass, specifically changes in body mass following physical activity, is perhaps the most feasible and commonly used method to assess hydration status. In many instances, the athlete is weighed prior to the start of a session with a goal of keeping total water losses to <2% of total body mass (Sawka et al., 2007). When more reliable information is desired, a 3-day baseline body mass can be used to establish daily fluctuations of fluid by deeming any changes from that baseline fluctuations in hydration status. One limitation to this method is that many athletes do not complete daily or 3-day baseline assessments in a euhydrated state, resulting in an underestimation of a euhydrated baseline body mass. This not only has implications for assessing hydration status prior to exercise but also when prescribing fluid to prevent >2% body mass loss. The following data provides an example of this concept using data published in a previous study (Benjamin et al., 2021b).

**FIGURE 3:**
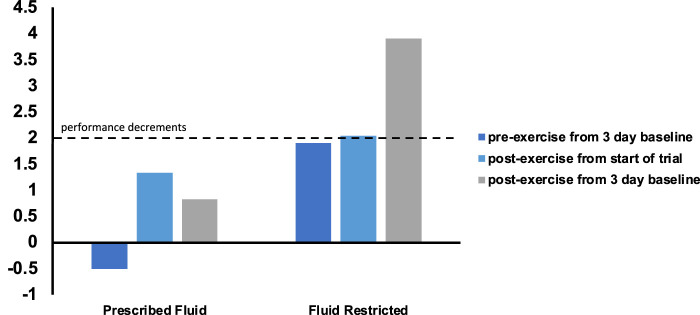
Body mass loss and “true” hydration status (Benjamin et al., 2021b).

In the Figure 3, participants were instructed to arrive at 3-day baseline assessments first thing in the morning euhydrated (Benjamin et al., 2021b). If their USG>1.025, participants were required to reschedule the visit and repeat the 3-day baseline (Benjamin et al., 2021b). Following the baseline, participants completed experimental trials in which they were instructed to arrive following a fluid intake protocol (confirmed by USG<1.020) or following a fluid restriction protocol (Benjamin et al., 2021b). If post-exercise body mass had not been compared to 3 days baseline body mass when calculating body mass loss, it would appear that the participant was not above the 2% body mass loss following exercise because the difference from the start of the trial was so small. Therefore, body mass loss does not indicate % dehydration in this case; for athletes who start exercise in a dehydrated state, this scenario is likely to result in the athlete not being aware of their true hydration status.

**FIGURE 4:**
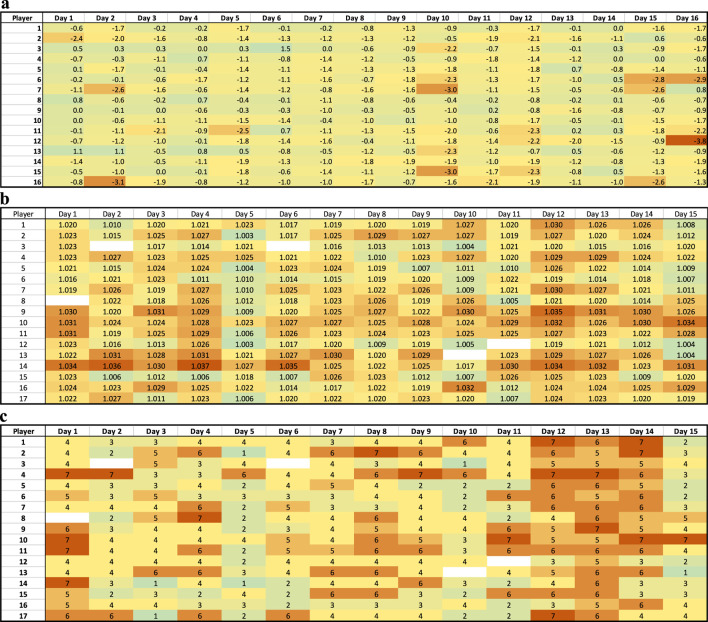
Individual variabilities of hydration status measured by **(a)** body mass loss, **(b)** urine specific gravity, **(c)** urine color in athletes. Urine was assessed before training and games and body mas was measured before and after (Sekiguchi et al., 2019).

This idea can be visualized from data collected from a collegiate soccer team over the course of pre-season (Sekiguchi et al., 2019). Figure 4 demonstrates that day-to-day fluctuations in body mass loss from training are relatively mild and rarely exceed 2%. However, if this is the only method used to assess hydration, athletes who are dehydrated may begin practice in a deficit. Therefore, those individuals require much less body mass loss to meet the 2%–3% body mass loss that is associated with negative performance implications. In this case, athletes who lost very little body mass in the session may think they are euhydrated but are in fact dehydrated because they started the session in a deficit.

### Fluid intake plans

Prior to discussing actionable solutions to drive a personalized approach to optimizing hydration strategies in sports and physical activity, it is important to highlight that current evidence-based recommendations are largely focused and isolated to the acute time periods surrounding sports participation (immediately prior to, during, and following sport) (Sawka et al., 2007; McDermott et al., 2017; Belval et al., 2019). It has been well established at the population level, that only 40%–60% of individuals meet daily fluid intake recommendations (Drewnowski et al., 2013b; Drewnowski et al., 2013a; Guelinckx et al., 2015a; Guelinckx et al., 2015b; Chang et al., 2016; Rosinger and Herrick, 2016). While the human body maintains normal body water at the day level through various hormonally-driven regulatory processes, not meeting daily fluid intake recommendations has been associated with adverse health outcomes and increased risks of morbidity and mortality (Thornton, 2014; Chang et al., 2016; Stookey et al., 2020; Dmitrieva et al., 2023). As hydration science continues to advance and evolve on topics related to optimizing personalized hydration strategies during sports, it is imperative that considerations surrounding daily fluid intake on health across the lifespan be incorporated into sports applications.

**FIGURE 5:**
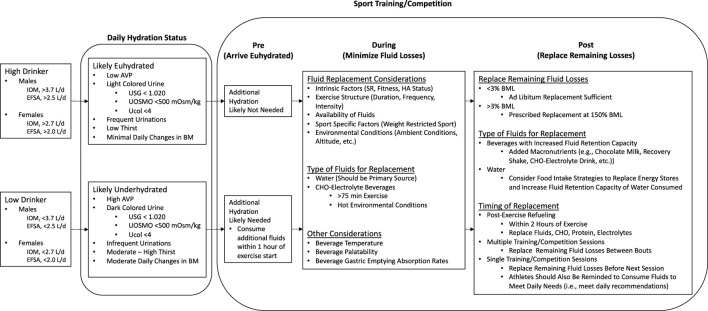
Individual, inter-individual, and organizational factors to develop individual fluid intake strategies. IOM, Institute of Medicine; EFSA, European Food Safety Authority; AVP, Arginine Vasopressin; USG, Urine Specific Gravity; UOSMO, Urine Osmolality, Ucol, Urine Color; BM, Body Mass; SR, Sweat Rate; HA, Heat Acclimation; CHO, Carbohydrate; BML, Body Mass Loss.

Current recommendations suggest that, in the context of sport participation, athletes arrive euhydrated, minimize fluid losses during exercise, and replace remaining losses following exercise (Sawka et al., 2007; McDermott et al., 2017; Belval et al., 2019). While this approach to maintaining proper hydration status is theoretically simplistic, there are many factors that may influence an athlete’s ability to attenuate dehydration-mediated adverse outcomes. Individual, inter-individual, and organizational factors can both independently and in combination influence one’s hydration needs, which must be incorporated into the development of individual fluid intake strategies (Figure 5) (Belval et al., 2019). While much of this discussion has focused on the adverse effects of dehydration, we must not neglect the potential concerns related to over-hydration and the risk of hyponatremia (Hew-Butler et al., 2015). By minimizing fluid losses based on fluid needs rather than prescribing a universal and absolute volume of fluid to consume, practitioners can thereby reduce the risk of hyponatremia.

In addition to the factors that influence one’s ability to maintain proper hydration status during sport, it would be remiss to not discuss beverage-specific considerations that should be incorporated into individual hydration plans. Beverage selection should consider factors such as gastric emptying/absorption rates, palatability, temperature, and availability (Baker and Jeukendrup, 2014). For example, water temperature between 15°C and 21°C is recommended to enhance the palatability (Sawka et al., 2007). Interestingly, colder water intake (e.g., 5°C) decreases the amount of fluid ingestion as well as sweating response (Hosseinlou et al., 2013). Also, the carbohydrate concentration should not exceed 8% to maintain gastric emptying rate (Jentjens et al., 2005; Wallis et al., 2005; Sawka et al., 2007). In the context of sports, water is the ideal beverage type to optimize hydration. However, the incorporation of other beverages such as carbohydrate-electrolyte beverages can be considered in circumstances where sports participation involves prolonged (>75 min) intense exercise, especially in hot environmental conditions.

Other considerations surrounding optimal beverage composition pertain to the timing of exercise. Following exercise, it is not uncommon for individuals to remain in a net-negative body water state (e.g., dehydration) which is often driven by factors such as voluntary hydration (Greenleaf, 1992). Given the importance of restoring remaining body water losses following exercise, and understanding the variability in sports schedules and timing of exercise, being intentional with selecting hydration beverages that have a greater fluid retention capacity (Maughan et al., 2016; Clarke et al., 2019; Bechke et al., 2022), may serve as an advantageous solution to negate the potential compounding effects of dehydration (Godek et al., 2005b; Godek et al., 2005a).

With the continued study of hydration science, future directions must involve approaches that address acute and longer-term impacts of fluid intake on health and performance. Not only is it important to identify and optimize sport-specific hydration strategies (e.g., team sport-based vs. individual sport-based strategies, age-related strategies, etc.), but also important to identify hydration strategies that can be implemented to drive healthy behavior changes.

### Variability and individualization of the sweating response and its components

It is well established that hydration status, before and during sport, properly managing fluid intake strategies is essential to minimize the adverse health and performance outcomes related to dehydration. The addition of environmental heat stress further exacerbates the detrimental effects of dehydration on physiologic function during exercise (Buono and Wall, 2000; Adams et al., 2014), thus developing effective strategies for the assessment of fluid losses and subsequent fluid needs is important. To adequately replace one’s fluid losses during exercise, it is critical to understand the mechanism through which these losses are derived (sweating), the composition of that fluid lost, and the factors that influence its rate and composition. A variety of factors influence the control of one’s sweat rate and sweat composition such as heat acclimatization status (Pandolf et al., 1988; Nielsen et al., 1997; Périard et al., 2015), level of training (Buono and Sjoholm, 1988), hydration status (Sawka et al., 1985), environmental conditions (Cleary et al., 2014), altitude/hypoxia (Kolka et al., 1987), clothing (McLellan et al., 1992; Havenith, 1999), body mass (Dennis and Noakes, 1999; Buresh et al., 2005), biologic sex (Gagnon and Kenny, 2011), circadian rhythm (Stephenson and Kolka, 1988), maturation (Inbar et al., 2004), age (Kenney and Fowler, 1988), and even diet (Armstrong et al., 1985). It is critical to understand that each of these factors contributes to sweat rate and composition in their own dynamic and individualized manner (Baker, 2017), thus precise measurement at a given “snapshot in time” is critical to the interpretation and clinical application of sweat measurement.

Sweat is comprised primarily of water and sodium chloride (NaCl), but is also comprised of other micronutrients such as potassium (K^+^), calcium, magnesium, iron, zinc, and copper (Baker, 2019). Sodium (Na^+^) is the largest constituent of sweat and thus is the most reported in the literature with chloride (Cl^−1^) following closely. Individual variation of the concentration of Na^+^ has been reported previously across a variety of sports (Baker et al., 2016; Baker et al., 2022; Davis et al., 2016; Barnes et al., 2019; Rollo et al., 2021), and commonly ranges from 10 to 90 mmol⋅L^−1^ for regional sweat and 20–80 mmol⋅L^−1^ for whole-body sweat measures (Baker et al., 2016; Barnes et al., 2019), however, Na^+^ varies with intensity by up to ∼150% at the highest intensities (Baker et al., 2019). Sodium replacement is important to achieve full fluid balance restoration, especially athletes who perform events impacted by hydration status (Casa et al., 2010; Cheuvront and Kenefick, 2014; Adams et al., 2017; Benjamin et al., 2021b; Racinais et al., 2021). Also, sodium increases glucose absorption in the small intestine (Shirreffs et al., 2004). Whole-body Cl^−1^ measures often range from 20–70 mmol⋅L^−1^ with the final levels of Na and Cl being primarily determined by the extent of the reabsorption of Na by the duct itself (Shirreffs and Maughan, 1997). For these reasons, it is difficult to determine an athlete’s individual needs for optimal electrolyte replacement during one’s training or macro-cycle and even more so, during or after individual exercise sessions, without precise measures or techniques.

### Whole-body sweat electrolyte testing methods

A variety of sweat testing measures are utilized in sports science to determine the composition of the sweat and the volume of electrolytes lost. Two of the most utilized are the whole body washdown (WBW) technique and the regional absorbent sweat patch prediction technique. The WBW technique is arguably still considered the “gold standard” measure for determining whole-body sweat electrolyte concentration and was first developed by Dill et al. (1933) and refined by Shirreffs and Maughan (1997). The methods of the WBW are described in detail by Armstrong and Casa in 2009 (Armstrong and Casa, 2009) and are commonly used in laboratory settings where the proper equipment is available. That said, the body of knowledge in the area estimation of whole body electrolyte losses via regional absorbent sweat patch prediction by Baker et al. (2020) has emerged as a more field expedient and practical measure (Barnes et al., 2019). This method was once determined to overestimate the actual electrolyte concentrations and whole-body losses (Shirreffs and Maughan, 1997), but has now been cross-validated with predicted whole-body sweat Na^+^ from all sites within a mean bias of 0–5 mmol⋅L^−1^ and within a 95% LOA of ±12–17 mmol⋅L^−1^ compared with measured whole-body sweat Na^+^ (Baker et al., 2020). Regardless of the method used to assess whole-body electrolyte losses, the issues of individualization and variability remain. With variabilities in the amount of Na^+^, it necessitates the need for precise individualized recommendations for electrolyte replenishment during and post-exercise in an effort to maintain normal blood Na^+^ levels and to avoid hyponatremia, especially during long duration events at lower intensities.

## Sports science practical application

**FIGURE 6:**
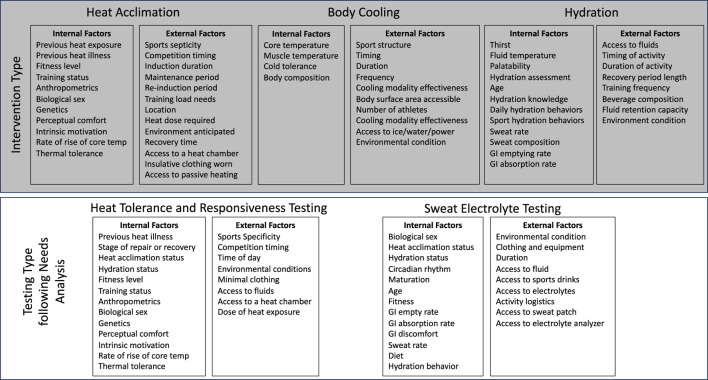
Factors to consider when determining and implementing individualized heat mitigation strategies.

**FIGURE 7:**
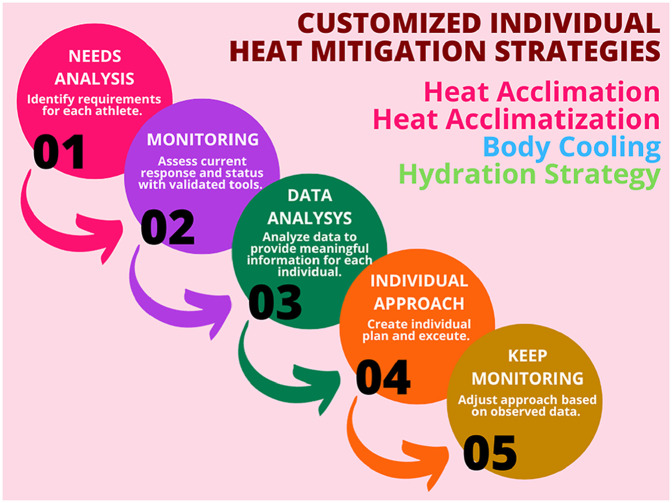
Conceptual process of evidence-based individual heat mitigation approach.

Considering individual variability for heat acclimation and heat acclimatization, various body cooling strategies, and hydration assessment/fluid replacement is important to maximize effects of these strategies, which lead to better performance and health outcomes. There are many factors to consider (Figure 6), and comprehensive approaches are required. The evidenced-informed decision is critical when making an individual approach, and data will help to make decisions effectively (Figure 7). Therefore, the first step will be the needs analysis and measuring athlete’s response and status by validated and reliable tools. It is important to consider what information can be obtained from variables before monitoring. Then, data will be analyzed to provide meaningful information from collected data. Traditional group analysis might not be appropriate here as the response is varied between each athlete (Rhea, 2004; Ward et al., 2018). Also, the approach to individual sports and team sports are different due to factors, such as the number of target competitions, frequency of the competitions, the duration of training period, and the number of athletes in the team. After understanding athlete status, an individual approach can be taken based on the goals and needs of each athlete. This process can be constantly repeated, and it is important to keep adjusting the approach based on observed data as it is useful information to check if the approach is effective. As summarized in Table 2, each strategy requires specific considerations when individualizing the plan.

**TABLE 2:** Specific considerations when creating an individualized plan.

Heat mitigation strategy	Specific considerations to individualization
Heat Acclimation Heat Acclimatization	• Select exercise modality (training and testing) based on sports specificity • Adjust intensity and volume based on individual responses to heat exposure • Decide optimal length based on individual responses to maximize effects but minimize fatigue • Select the frequency of intermittent heat training based on individual response and schedule of sports • Monitor individual workload in sports activity to maintain the balance between heat acclimation/acclimatization and sports
Body Cooling	• Select cooling modality based on sports, timing, feasibility, cooling capacity, and individual characteristics • Decide cooling duration based on individual response to cooling and the feasibility • Monitor changes in performance and health outcomes resulting from the implemented cooling strategy • As these factors change, cooling modality and duration might be adjusted
Hydration	• Select hydration assessment methods fitting individual situations based on sports and feasibility • Interpret hydration assessment data appropriately • Determine day to day and sports specific fluid needs • Implement a fluid intake plan based on hydration assessment • As exercise factors (e.g., intensity, duration, type), environmental conditions, and individual characteristics change, the fluid intake plan needs to be adjusted

Limitations of individual approach might be the requirements of extra cost, human resources, and time depending on the situation. Therefore, it is also important to be flexible in the approach to take and find the best evidence-based solution in each scenario. However, this approach might be essential to maximize heat mitigation strategies, including decrease the negative impact of heat stress and dehydration, which could be one of the factors deciding the results of competition. Heat acclimation and heat acclimatization, various body cooling strategies, and hydration assessment/fluid replacement can improve exercise performance in a relatively easy and cost and time-efficient way (Sawka et al., 2007; Bongers et al., 2015; Tyler et al., 2016). It is important to emphasize that no matter what training the athlete performs if these factors are not considered on the game day, athletes’ performance might not reach their maximum. Future directions would be gathering evidence and information about how to take individual approaches and what outcomes can be observed by each approach.

## Examples of heat mitigation strategies based on specific scenarios

Examples of individualized heat mitigation plans are described for three different scenarios below. The heat mitigation strategies are different for each scenario based on factors, such as the type of sports, the period of the preparation, environmental conditions, and the situation of competition day. While individual sports have typically one target race/competition, team sports have multiple competitions throughout the season, which also leads to different approaches.

### Scenario 1: Kona triathlon world championship

A 35-year-old female athlete residing in the Southern Hemisphere will be competing in the 2025 Kona Triathlon World Championships, which is held in Kona, Hawaii in October. The expected environmental conditions are: ambient temperature, 33°C–24°C (90°F–75°F); relative humidity, 80%; water temperature, ∼27°C (∼80°C). The athlete will be traveling to Kona 7 days prior to the race from her home.

Individualized heat acclimation/acclimatization strategies:

•Perform 10–14 days of a heat acclimation protocol 4–6 weeks prior to race day (- 4 weeks from the race).
  - 90 min exercise (run or bike) in an environmental controlled chamber (33°C and 50% humidity).
  - Try to achieve core temperature above 38.5°C for a minimum of 60 min during each session.
  - Humidity can be gradually increased to 80% over the course of the acclimation period to mimic the expected racing conditions.
•Modify training volume and intensity based on heat acclimation training in order not to accumulate fatigue, and in alignment with the current training regimen.
•Perform intermittent heat training once or twice a week for 2 weeks prior to travel to Kona (−3 and −2 weeks from the race).
  - Exercise protocol can be the same as a heat acclimation session.
  - This session can be performed as part of “easy” training day.
•After arriving in Kona, perform one training session (∼90 min of exercise) at 33°C and 80% humidity. The rest of the training can be done when the environmental conditions are cooler to adequately prepare for the race and optimize the potential for peak performance.

Individualized body cooling strategies for competition:

•Note: All planned cooling strategies should be practiced prior to race day in order to limit the implementation of anything “new” to the athlete on the day of the race itself.
•Prepare and load a squeeze bottle filled with ice and water at the swim-to-bike transition area; this bottle is prepared for water dousing during the bike segment as this strategy can be used to maximize both convective and evaporative heat loss during cycling (>30 km⋅h^−1^). If possible, the water bottle is insulated to keep fluid chilled.
•Throughout the bike course, there will be aid stations where water bottles (plastic bottles with caps) are provided. Considerations need to be made to determine the amount of water to be used for hydration vs. cooling/dousing.
•Prepare an insulated water bottle at the bike-to-run transition area; during ice slurry or chilled fluid at the transition.
•The run course will also have aid stations with water; during this segment of the race, water is provided in cups. Use water dousing strategies during the run segment. If aid stations have ice, consider packing jersey compartments with ice.
•Wet clothes may cause chafing–apply tape and/or lubricants to protect areas where friction may occur.

Individualized hydration strategy

•During training, determine daily fluid needs to ensure euhydration day-to-day. Using daily, and first morning, assessments of body weight, urine color, and thirst are useful and low-cost strategies to determine the adequacy of water intake. Body weight loss >1%, dark colored urine, and/or increased thirst perception indicates inadequacy of fluid intake the day prior.
•Assess sweat rate during training at race pace for bike and run disciplines to determine fluid losses and associated fluid needs to minimize fluid losses. Ideally, this should be assessed at multiple times throughout the training cycle, including in environmental conditions that are expected on race day.
•The race day hydration strategy should include considerations for both fluid losses as well as overall nutrition/energy needs. Identifying beverages containing carbohydrates and electrolytes that can be tolerated during exercise should be explored and be supplemented in addition to water.
•During race day, ensure that the practiced hydration strategy is followed. This includes what occurs across disciplines as well as during the swim - bike, and bike - run transitions.
•Determining post-race fluid losses and replenishing remaining losses in the 24 h following the race is important to aid recovery.

### Scenario 2: international soccer tournament

A national soccer team will be participating in an international soccer tournament that is scheduled to take place during the summer months (average 29°C–32°C). Players are currently in their normal off-season training cycle and practices take place in a relatively cooler geographical location (average 20°C–22°C). The team will be traveling to the tournament location 14 days prior to the first match for the team’s pre-tournament training camp to adjust for training in the respective environmental conditions and to assist in overcoming jet lag.

Individualized heat acclimation/acclimatization strategies:

•Prior to international travel to the team’s training camp: perform passive heat acclimation (e.g., 30–45 min of sauna or hot bath following daily training sessions).
•During the first week of the international training camp prior to the start of the tournament, training needs to be performed in mild environmental conditions when workload is relatively high but can be done in relatively high environmental conditions when the workload is low to maintain heat exposure but not accumulate fatigue.
•During the second week of training camp, the volume needs to be decreased gradually so does heat exposure to prepare for the first game at the good overall condition, which includes fitness and heat adaptation Table 3.

**TABLE 3:** Training load and heat exposure magnitude.

Day from 1st game	−14	−13	−12	−11	−10	−9	−8	−7	−6	−5	−4	−3	−2	−1
Training Load	L	M/H	M	Off	H	L	M/H	M	Off	M	M	Off	M	L
Heat Exposure Magnitude	M	H	M	Off	H	M	H	M	Off	M	M	Off	L	L

L: low, M: medium, H: high

Individualized body cooling strategies for competition:

•Pre-game warm-up session: prepare ice slurry for all athletes (7.5 g per kilogram in body weight) and cooling vests for starting members.
•Towel off sweat, drink chilled fluid and/or ice slurry, and partial body immersion (forearm or lower leg) during half-time. Prepare a bucket filled with ice and water per athlete. If partial body immersion is not feasible, use cooling vests.
•Post-game recovery using whole-body cold-water immersion or contrast bath ending with cold water immersion.

Individualized hydration strategies

• During training, determine daily fluid needs to ensure euhydration day-to-day. Using daily, and first morning, assessments of body weight, urine color, and thirst are useful and low-cost strategies to determine the adequacy of water intake. Body weight loss >1%, dark colored urine, and/or increased thirst perception indicates inadequacy of fluid intake the day prior.
•Ensure an euhydrated arrival prior to each competition match.
•Use available break times (e.g., half time, schedule cooling breaks when environmental conditions require implementation of a cooling break) to minimize fluid losses.
•Fluid replacement should include water and could be supplemented by a carbohydrate electrolyte beverage, especially when competition is occurring in hot environmental conditions and the athlete is participating in the entire match.

### Scenario 3: American football

A National Football League team is commencing their fall training camp with the camp being held at a nearby university July 24 - August 11th. All training sessions occur outdoors between 1,500–1,700 h daily and takes place on a natural grass playing field. Ambient temperatures will range from 26–30°C with a relative humidity being approximately 80%.

Individualized heat acclimation/acclimatization strategies:

•2 weeks prior to the start of preseason, an athlete starts performing 10–14 heat acclimation sessions in the same environmental condition as preseason training is performed.
•Exercise can be 60–90 min moderate exercise to maintain core temperature above 38.5°C for ∼60 min.
•The duration of exercise can be gradually increased as the session goes.
•No heat acclimation session from 2 days prior to the start of preseason to recover from heat acclimation. Once preseason starts, follow league mandated heat acclimatization protocol.
•After the preseason, once a week of intermittent heat training is performed to maintain adaptations throughout the competitive season. This can be done as part of aerobic exercise training day for the recovery.

Individualized body cooling strategies during pre-season football practices:

•Prepare a cooling zone with a tent (shade), cold-water immersion bath, ice chest filled with ice, chilled fluid, mist fan, and ice towels. The size of the cooling area and amount of equipment (e.g., misting fans, ice towels, etc.) should be large enough to cover all members of a given unit (e.g., offensive players) at the same time.
•Helmets should be taken off during water breaks to maximize evaporative heat loss. Use combinations of water dousing, ice towels, misting fan, and chilled fluid intake for per-cooling during practice sessions.
•Post-training recovery using whole-body cold-water immersion or contrast bath ending with cold water immersion.

Individualized hydration strategies

•During training, determine daily fluid needs to ensure euhydration day-to-day. Using daily, and first morning, assessments of body weight, urine color, and thirst are useful and low-cost strategies to determine the adequacy of water intake. Body weight loss >1%, dark colored urine, and/or increased thirst perception indicates inadequacy of fluid intake the day prior.
•Ensure an euhydrated arrival prior to each training session.
•Consume fluids during training and during breaks to minimize fluid losses. Fluids should primarily be water, however carbohydrate electrolyte beverages can also be incorporated, especially during prolonged (>75 min) and intense training in hot environmental conditions.
•Organizationally, the team should provide unlimited access to fluids to all athletes during training. Similarly, the team should be adequately staffed to ensure access to fluid is unlimited.

## References

[B1] Adams J. D. Sekiguchi Y. Seal A. Suh H.-G. Sprong C. Jansen L. (2017). Dehydration impairs exercise performance independent of thirst perception: a blinded study. Med. and Sci. Sports and Exerc. 49, 833. 10.1249/01.mss.0000519236.20188.7b 29509643

[B2] Adams W. M. Anderson T. Zaplatosch M. E. Cheuvront S. N. Kenefick R. W. Yates B. A. (2024). Utility of body weight, urine color, and thirst perception (WUT) in determining hydration in young adults. Med. Sci. Sports Exerc 56, 2404–2412. 10.1249/MSS.0000000000003514 38967358

[B3] Adams W. M. Ferraro E. M. Huggins R. A. Casa D. J. (2014). Influence of body mass loss on changes in heart rate during exercise in the heat: a systematic review. J. Strength Cond. Res. 28, 2380–2389. 10.1519/JSC.0000000000000501 24736771

[B4] Adams W. M. Hosokawa Y. Casa D. J. (2016). Body-cooling paradigm in sport: maximizing safety and performance during competition. J. Sport Rehabil. 25, 382–394. 10.1123/jsr.2015-0008 27632890

[B5] Alhadad S. B. Tan P. M. S. Lee J. K. W. (2019). Efficacy of heat mitigation strategies on core temperature and endurance exercise: a meta-analysis. Front. Physiol. 10, 71. 10.3389/fphys.2019.00071 30842739 PMC6391927

[B6] Alkemade P. Gerrett N. Eijsvogels T. M. H. Daanen H. A. M. (2021). Individual characteristics associated with the magnitude of heat acclimation adaptations. Eur. J. Appl. Physiol. 121, 1593–1606. 10.1007/s00421-021-04626-3 33646425 PMC8144163

[B7] Armstrong L. E. (2007). Assessing hydration status: the elusive gold standard. J. Am. Coll. Nutr. 26, 575S-584S–584S. 10.1080/07315724.2007.10719661 17921468

[B8] Armstrong L. E. Casa D. J. (2009). Methods to evaluate electrolyte and water turnover of athletes. Athl. Train. and Sports Health Care 1, 169–179. 10.3928/19425864-20090625-06

[B9] Armstrong L. E. Costill D. L. Fink W. J. Bassett D. Hargreaves M. Nishibata I. (1985). Effects of dietary sodium on body and muscle potassium content during heat acclimation. Eur. J. Appl. Physiol. 54, 391–397. 10.1007/BF02337183 4065126

[B10] Armstrong L. E. Hubbard R. W. DeLUCA J. P. Christensen E. L. (1987). Heat acclimatization during summer running in the northeastern United States. Med. and Sci. Sports and Exerc. 19, 131–136. 10.1249/00005768-198704000-00011 3574045

[B11] Armstrong L. E. Kavouras S. A. (2019). Thirst and drinking paradigms: evolution from single factor effects to brainwide dynamic networks. Nutrients 11, 2864. 10.3390/nu11122864 31766680 PMC6950074

[B12] Armstrong L. E. Maresh C. M. (1991). The induction and decay of heat acclimatisation in trained athletes. Sports Med. 12, 302–312. 10.2165/00007256-199112050-00003 1763248

[B13] Baillot M. Hue O. Tran T. T. Antoine-Jonville S. (2021). Neuromuscular activity during cycling performance in hot/dry and hot/humid conditions. Life (Basel) 11, 1149. 10.3390/life11111149 34833025 PMC8623245

[B14] Baker L. B. (2017). Sweating rate and sweat sodium concentration in athletes: a review of methodology and intra/interindividual variability. Sports Med. 47, 111–128. 10.1007/s40279-017-0691-5 28332116 PMC5371639

[B15] Baker L. B. (2019). Physiology of sweat gland function: the roles of sweating and sweat composition in human health. Temp. (Austin) 6, 211–259. 10.1080/23328940.2019.1632145 PMC6773238 31608304

[B16] Baker L. B. Barnes K. A. Anderson M. L. Passe D. H. Stofan J. R. (2016). Normative data for regional sweat sodium concentration and whole-body sweating rate in athletes. J. Sports Sci. 34, 358–368. 10.1080/02640414.2015.1055291 26070030

[B17] Baker L. B. De Chavez P. J. D. Ungaro C. T. Sopeña B. C. Nuccio R. P. Reimel A. J. (2019). Exercise intensity effects on total sweat electrolyte losses and regional vs. whole-body sweat [Na+], [Cl−], and [K+]. Eur. J. Appl. Physiol. 119, 361–375. 10.1007/s00421-018-4048-z 30523403 PMC6373370

[B18] Baker L. B. Jeukendrup A. E. (2014). Optimal composition of fluid-replacement beverages. Compr. Physiol. 4, 575–620. 10.1002/cphy.c130014 24715561

[B19] Baker L. B. King M. A. Keyes D. M. Brown S. D. Engel M. D. Seib M. S. (2022). Sweating rate and sweat chloride concentration of elite male basketball players measured with a wearable microfluidic device versus the standard absorbent patch method. Int. J. Sport Nutr. Exerc Metab. 32, 342–349. 10.1123/ijsnem.2022-0017 35477899

[B20] Baker L. B. Nuccio R. P. Reimel A. J. Brown S. D. Ungaro C. T. De Chavez P. J. D. (2020). Cross-validation of equations to predict whole-body sweat sodium concentration from regional measures during exercise. Physiol. Rep. 8, e14524. 10.14814/phy2.14524 32748563 PMC7399373

[B21] Barnes K. A. Anderson M. L. Stofan J. R. Dalrymple K. J. Reimel A. J. Roberts T. J. (2019). Normative data for sweating rate, sweat sodium concentration, and sweat sodium loss in athletes: an update and analysis by sport. J. Sports Sci. 37, 2356–2366. 10.1080/02640414.2019.1633159 31230518

[B22] Bechke E. E. Zaplatosch M. E. Choi J.-Y. Adams W. M. (2022). Utility of an isotonic beverage on hydration status and cardiovascular alterations. Nutrients 14, 1286. 10.3390/nu14061286 35334943 PMC8953172

[B23] Belval L. N. Hosokawa Y. Casa D. J. Adams W. M. Armstrong L. E. Baker L. B. (2019). Practical hydration solutions for sports. Nutrients 11, 1550. 10.3390/nu11071550 31324008 PMC6682880

[B24] Benjamin C. L. Sekiguchi Y. Armstrong L. E. Manning C. N. Struder J. F. Butler C. R. (2021a). The efficacy of weekly and bi-weekly heat training to maintain the physiological benefits of heat acclimation. J. Sci. Med. Sport S1440-2440 (21), 255–260. 10.1016/j.jsams.2021.10.006 34750069

[B25] Benjamin C. L. Sekiguchi Y. Morrissey M. C. Butler C. R. Filep E. M. Steans R. L. (2021b). The effects of hydration status and ice-water dousing on physiological and performance indices during a simulated soccer match in the heat. J. Sci. Med. Sport 0, 723–728. 10.1016/j.jsams.2021.05.013 34140229

[B26] Benjamin C. L. Sekiguchi Y. Struder J. F. Szymanski M. R. Manning C. N. Grundstein A. J. (2021d). Heat acclimation following heat acclimatization elicits additional physiological improvements in male endurance athletes. Int. J. Environ. Res. Public Health 18, 4366. 10.3390/ijerph18084366 33924138 PMC8074339

[B27] Bongers C. C. W. G. Hopman M. T. E. Eijsvogels T. M. H. (2017). Cooling interventions for athletes: an overview of effectiveness, physiological mechanisms, and practical considerations. Temp. (Austin) 4, 60–78. 10.1080/23328940.2016.1277003 PMC5356217 28349095

[B28] Bongers C. C. W. G. Thijssen D. H. J. Veltmeijer M. T. W. Hopman M. T. E. Eijsvogels T. M. H. (2015). Precooling and percooling (cooling during exercise) both improve performance in the heat: a meta-analytical review. Br. J. Sports Med. 49, 377–384. 10.1136/bjsports-2013-092928 24747298

[B29] Bradley L. J. Miller K. C. Wiese B. W. Novak J. R. (2019). Precooling’s effect on American football skills. J. Strength Cond. Res. 33, 2616–2621. 10.1519/JSC.0000000000003330 31425459

[B30] Buchheit M. Racinais S. Bilsborough J. C. Bourdon P. C. Voss S. C. Hocking J. (2013). Monitoring fitness, fatigue and running performance during a pre-season training camp in elite football players. J. Sci. Med. Sport 16, 550–555. 10.1016/j.jsams.2012.12.003 23332540

[B31] Buono M. J. Sjoholm N. T. (1988). Effect of physical training on peripheral sweat production. J. Appl. Physiol. 65, 811–814. 10.1152/jappl.1988.65.2.811 3170430

[B32] Buono M. J. Wall A. J. (2000). Effect of hypohydration on core temperature during exercise in temperate and hot environments. Pflugers Arch. 440, 476–480. 10.1007/s004240000298 10954335

[B33] Buresh R. Berg K. Noble J. (2005). Heat production and storage are positively correlated with measures of body size/composition and heart rate drift during vigorous running. Res. Q. Exerc. Sport 76, 267–274. 10.1080/02701367.2005.10599298 16270704

[B34] Cao Y. Lei T.-H. Wang F. Yang B. Mündel T. (2022). Head, face and neck cooling as per-cooling (cooling during exercise) modalities to improve exercise performance in the heat: a narrative review and practical applications. Sports Med. Open 8, 16. 10.1186/s40798-022-00411-4 35092517 PMC8800980

[B35] Casa D. J. Stearns R. L. Lopez R. M. Ganio M. S. McDermott B. P. Walker Yeargin S. (2010). Influence of hydration on physiological function and performance during trail running in the heat. J. Athl. Train. 45, 147–156. 10.4085/1062-6050-45.2.147 20210618 PMC2838466

[B36] Casadio J. R. Kilding A. E. Cotter J. D. Laursen P. B. (2017). From lab to real world: heat acclimation considerations for elite athletes. Sports Med. 47, 1467–1476. 10.1007/s40279-016-0668-9 28035584

[B37] Chalmers S. Esterman A. Eston R. Bowering K. J. Norton K. (2014). Short-term heat acclimation training improves physical performance: a systematic review, and exploration of physiological adaptations and application for team sports. Sports Med. 44, 971–988. 10.1007/s40279-014-0178-6 24817609

[B38] Chang T. Ravi N. Plegue M. A. Sonneville K. R. Davis M. M. (2016). Inadequate hydration, BMI, and obesity among US adults: NHANES 2009-2012. Ann. Fam. Med. 14, 320–324. 10.1370/afm.1951 27401419 PMC4940461

[B39] Cheuvront S. N. Kenefick R. W. (2014). Dehydration: physiology, assessment, and performance effects. Compr. Physiol. 4, 257–285. 10.1002/cphy.c130017 24692140

[B40] Chou T.-H. Allen J. R. Hahn D. Leary B. K. Coyle E. F. (2018). Cardiovascular responses to exercise when increasing skin temperature with narrowing of the core-to-skin temperature gradient. J. Appl. Physiol. 125, 697–705. 10.1152/japplphysiol.00965.2017 29745802

[B41] Clarke M. M. Stanhewicz A. E. Wolf S. T. Cheuvront S. N. Kenefick R. W. Kenney W. L. (2019). A randomized trial to assess beverage hydration index in healthy older adults. Am. J. Clin. Nutr. 109, 1640–1647. 10.1093/ajcn/nqz009 31051498 PMC6537935

[B42] Cleary M. A. Toy M. G. Lopez R. M. (2014). Thermoregulatory, cardiovascular, and perceptual responses to intermittent cooling during exercise in a hot, humid outdoor environment. J. Strength Cond. Res. 28, 792–806. 10.1519/JSC.0b013e3182a20f57 23897015

[B43] Corbett J. Rendell R. A. Massey H. C. Costello J. T. Tipton M. J. (2018). Inter-individual variation in the adaptive response to heat acclimation. J. Therm. Biol. 74, 29–36. 10.1016/j.jtherbio.2018.03.002 29801640

[B44] Daanen H. A. M. Racinais S. Périard J. D. (2018). Heat acclimation decay and Re-induction: a systematic review and meta-analysis. Sports Med. 48, 409–430. 10.1007/s40279-017-0808-x 29129022 PMC5775394

[B45] Davis J. K. Baker L. B. Barnes K. Ungaro C. Stofan J. (2016). Thermoregulation, fluid balance, and sweat losses in American football players. Sports Med. 46, 1391–1405. 10.1007/s40279-016-0527-8 27071988

[B46] Dennis S. C. Noakes T. D. (1999). Advantages of a smaller bodymass in humans when distance-running in warm, humid conditions. Eur. J. Appl. Physiol. Occup. Physiol. 79, 280–284. 10.1007/s004210050507 10048634

[B47] Dill D. B. Jones B. F. Edwards H. T. Oberg S. A. (1933). Salt economy in extreme dry heat. J. Biol. Chem. 100, 755–767. 10.1016/s0021-9258(18)75949-8

[B48] Distefano L. J. Casa D. J. Vansumeren M. M. Karslo R. M. Huggins R. A. Demartini J. K. (2013). Hypohydration and hyperthermia impair neuromuscular control after exercise. Med. Sci. Sports Exerc 45, 1166–1173. 10.1249/MSS.0b013e3182805b83 23274594

[B49] Dmitrieva N. I. Gagarin A. Liu D. Wu C. O. Boehm M. (2023). Middle-age high normal serum sodium as a risk factor for accelerated biological aging, chronic diseases, and premature mortality. eBioMedicine 0, 104404. 10.1016/j.ebiom.2022.104404 PMC9873684 36599719

[B50] Douzi W. Dugué B. Vinches L. Al Sayed C. Hallé S. Bosquet L. (2019). Cooling during exercise enhances performances, but the cooled body areas matter: a systematic review with meta-analyses. Scand. J. Med. Sci. Sports 29, 1660–1676. 10.1111/sms.13521 31340407

[B51] Drewnowski A. Rehm C. D. Constant F. (2013a). Water and beverage consumption among adults in the United States: cross-sectional study using data from NHANES 2005-2010. BMC Public Health 13, 1068. 10.1186/1471-2458-13-1068 24219567 PMC3840570

[B52] Drewnowski A. Rehm C. D. Constant F. (2013b). Water and beverage consumption among children age 4-13y in the United States: analyses of 2005-2010 NHANES data. Nutr. J. 12, 85. 10.1186/1475-2891-12-85 23782914 PMC3698018

[B53] Drust B. Rasmussen P. Mohr M. Nielsen B. Nybo L. (2005). Elevations in core and muscle temperature impairs repeated sprint performance. Acta Physiol. Scand. 183, 181–190. 10.1111/j.1365-201X.2004.01390.x 15676059

[B54] Gagnon D. Kenny G. P. (2011). Sex modulates whole-body sudomotor thermosensitivity during exercise. J. Physiol. 589, 6205–6217. 10.1113/jphysiol.2011.219220 22005684 PMC3286696

[B55] Galloway S. D. Maughan R. J. (1997). Effects of ambient temperature on the capacity to perform prolonged cycle exercise in man. Med. Sci. Sports Exerc 29, 1240–1249. 10.1097/00005768-199709000-00018 9309637

[B56] Godek S. F. Bartolozzi A. R. Godek J. J. (2005a). Sweat rate and fluid turnover in American football players compared with runners in a hot and humid environment. Br. J. Sports Med. 39, 205–211. 10.1136/bjsm.2004.011767 15793087 PMC1725187

[B57] Godek S. F. Godek J. J. Bartolozzi A. R. (2005b). Hydration status in college football players during consecutive days of twice-a-day preseason practices. Am. J. Sports Med. 33, 843–851. 10.1177/0363546504270999 15827364

[B58] Godek S. F. Morrison K. E. Scullin G. (2017). Cold-water immersion cooling rates in football linemen and cross-country runners with exercise-induced hyperthermia. J. Athl. Train. 52, 902–909. 10.4085/1062-6050-52.7.08 28937782 PMC5687234

[B59] González-Alonso J. Mora-Rodríguez R. Below P. R. Coyle E. F. (1995). Dehydration reduces cardiac output and increases systemic and cutaneous vascular resistance during exercise. J. Appl. Physiol. 79, 1487–1496. 10.1152/jappl.1995.79.5.1487 8594004

[B60] González-Alonso J. Mora-Rodríguez R. Below P. R. Coyle E. F. (1997). Dehydration markedly impairs cardiovascular function in hyperthermic endurance athletes during exercise. J. Appl. Physiol. 82, 1229–1236. 10.1152/jappl.1997.82.4.1229 9104860

[B61] González-Alonso J. Teller C. Andersen S. L. Jensen F. B. Hyldig T. Nielsen B. (1999). Influence of body temperature on the development of fatigue during prolonged exercise in the heat. J. Appl. Physiol. 86, 1032–1039. 10.1152/jappl.1999.86.3.1032 10066720

[B62] Greenleaf J. E. (1992). Problem: thirst, drinking behavior, and involuntary dehydration. Med. Sci. Sports Exerc 24, 645–656. 10.1249/00005768-199206000-00007 1602937

[B63] Guelinckx I. Ferreira-Pêgo C. Moreno L. A. Kavouras S. A. Gandy J. Martinez H. (2015a). Intake of water and different beverages in adults across 13 countries. Eur. J. Nutr. 54 (Suppl. 2), 45–55. 10.1007/s00394-015-0952-8 26072214 PMC4473281

[B64] Guelinckx I. Iglesia I. Bottin J. H. De Miguel-Etayo P. González-Gil E. M. Salas-Salvadó J. (2015b). Intake of water and beverages of children and adolescents in 13 countries. Eur. J. Nutr. 54 (Suppl. 2), 69–79. 10.1007/s00394-015-0955-5 26072216 PMC4473084

[B65] Havenith G. (1999). Heat balance when wearing protective clothing. Ann. Occup. Hyg. 43, 289–296. 10.1016/s0003-4878(99)00051-4 10481628

[B66] Hew-Butler T. Rosner M. H. Fowkes-Godek S. Dugas J. P. Hoffman M. D. Lewis D. P. (2015). Statement of the third international exercise-associated hyponatremia consensus development conference, carlsbad, California, 2015. Clin. J. Sport Med. 25, 303–320. 10.1097/JSM.0000000000000221 26102445

[B67] Hosseinlou A. Khamnei S. Zamanlu M. (2013). The effect of water temperature and voluntary drinking on the post rehydration sweating. Int. J. Clin. Exp. Med. 6, 683–687. 24040477 PMC3762624

[B68] Hutchins K. P. Borg D. N. Bach A. J. E. Bon J. J. Minett G. M. Stewart I. B. (2021). Female (under) representation in exercise thermoregulation research. Sports Med. Open 7, 43. 10.1186/s40798-021-00334-6 34156570 PMC8219822

[B69] Inbar O. Morris N. Epstein Y. Gass G. (2004). Comparison of thermoregulatory responses to exercise in dry heat among prepubertal boys, young adults and older males. Exp. Physiol. 89, 691–700. 10.1113/expphysiol.2004.027979 15328309

[B70] Iwata R. Kawamura T. Hosokawa Y. Chang L. Suzuki K. Muraoka I. (2020). Differences between sexes in thermoregulatory responses and exercise time during endurance exercise in a hot environment following pre-cooling with ice slurry ingestion. J. Therm. Biol. 94, 102746. 10.1016/j.jtherbio.2020.102746 33292987

[B71] Jentjens R. L. P. G. Shaw C. Birtles T. Waring R. H. Harding L. K. Jeukendrup A. E. (2005). Oxidation of combined ingestion of glucose and sucrose during exercise. Metabolism 54, 610–618. 10.1016/j.metabol.2004.12.004 15877291

[B72] Jentjens R. L. P. G. Wagenmakers A. J. M. Jeukendrup A. E. (2002). Heat stress increases muscle glycogen use but reduces the oxidation of ingested carbohydrates during exercise. J. Appl. Physiol. 92, 1562–1572. 10.1152/japplphysiol.00482.2001 11896023

[B73] Jiang D. Yu Q. Liu M. Dai J. (2022). Effects of different external cooling placements prior to and during exercise on athletic performance in the heat: a systematic review and meta-analysis. Front. Physiol. 13, 1091228. 10.3389/fphys.2022.1091228 36703929 PMC9871495

[B74] Jones P. R. Barton C. Morrissey D. Maffulli N. Hemmings S. (2012). Pre-cooling for endurance exercise performance in the heat: a systematic review. BMC Med. 10, 166. 10.1186/1741-7015-10-166 23249542 PMC3568721

[B75] Keefe M. S. Luk H.-Y. Rolloque J.-J. Jiwan N. C. Sekiguchi Y. (2025). Hydration assessment in males and females using the WUT (weight, urine color, and thirst) Venn diagram compared to blood and urinary indices. Nutrients 17, 689. 10.3390/nu17040689 40005016 PMC11858619

[B76] Keefe M. S. Luk H.-Y. Rolloque J.-J. S. Jiwan N. C. McCollum T. B. Sekiguchi Y. (2024). The weight, urine colour and thirst Venn diagram is an accurate tool compared with urinary and blood markers for hydration assessment at morning and afternoon timepoints in euhydrated and free-living individuals. Br. J. Nutr. 131, 1181–1188. 10.1017/S000711452300274X 38012859 PMC10918520

[B77] Kenney W. L. Fowler S. R. (1988). Methylcholine-activated eccrine sweat gland density and output as a function of age. J. Appl. Physiol. 65, 1082–1086. 10.1152/jappl.1988.65.3.1082 3182477

[B78] Kerr Z. Y. Register-Mihalik J. K. Pryor R. R. Pierpoint L. A. Scarneo S. E. Adams W. M. (2019). The association between mandated preseason heat acclimatization guidelines and exertional heat illness during preseason high school American football practices. Environ. Health Perspect. 127, 47003. 10.1289/EHP4163 30969138 PMC6777902

[B79] Koenig F. S. Miller K. C. O’Connor P. Amaria N. (2022). Body anthropometric characteristics and rectal temperature cooling rates in women with hyperthermia. J. Athl. Train. 57, 464–469. 10.4085/1062-6050-225-20 35230443 PMC9205556

[B80] Kolka M. A. Stephenson L. A. Rock P. B. Gonzalez R. R. (1987). Local sweating and cutaneous blood flow during exercise in hypobaric environments. J. Appl. Physiol. 62, 2224–2229. 10.1152/jappl.1987.62.6.2224 3610918

[B81] Lemire B. B. Gagnon D. Jay O. Kenny G. P. (2009). Differences between sexes in rectal cooling rates after exercise-induced hyperthermia. Med. Sci. Sports Exerc 41, 1633–1639. 10.1249/MSS.0b013e31819e010c 19568196

[B82] Logan-Sprenger H. M. Heigenhauser G. J. F. Killian K. J. Spriet L. L. (2012). Effects of dehydration during cycling on skeletal muscle metabolism in females. Med. Sci. Sports Exerc 44, 1949–1957. 10.1249/MSS.0b013e31825abc7c 22543739

[B83] Lopez R. M. Casa D. J. Jensen K. A. DeMartini J. K. Pagnotta K. D. Ruiz R. C. (2011). Examining the influence of hydration status on physiological responses and running speed during trail running in the heat with controlled exercise intensity. J. Strength Cond. Res. 25, 2944–2954. 10.1519/JSC.0b013e318231a6c8 22024610

[B84] Lorenzo S. Halliwill J. R. Sawka M. N. Minson C. T. (2010). Heat acclimation improves exercise performance. J. Appl. Physiol. 109, 1140–1147. 10.1152/japplphysiol.00495.2010 20724560 PMC2963322

[B85] Marino F. E. (2002). Methods, advantages, and limitations of body cooling for exercise performance. Br. J. Sports Med. 36, 89–94. 10.1136/bjsm.36.2.89 11916888 PMC1724476

[B86] Maughan R. J. Watson P. Cordery P. A. Walsh N. P. Oliver S. J. Dolci A. (2016). A randomized trial to assess the potential of different beverages to affect hydration status: development of a beverage hydration index. Am. J. Clin. Nutr. 103, 717–723. 10.3945/ajcn.115.114769 26702122

[B87] Maunder E. Plews D. J. Merien F. Kilding A. E. (2020). Exercise intensity regulates the effect of heat stress on substrate oxidation rates during exercise. Eur. J. Sport Sci. 20, 935–943. 10.1080/17461391.2019.1674928 31566098

[B88] McDermott B. P. Anderson S. A. Armstrong L. E. Casa D. J. Cheuvront S. N. Cooper L. (2017). National athletic trainers’ association position statement: fluid replacement for the physically active. J. Athl. Train. 52, 877–895. 10.4085/1062-6050-52.9.02 28985128 PMC5634236

[B89] McLellan T. M. Meunier P. Livingstone S. (1992). Influence of a new vapor protective clothing layer on physical work tolerance times at 40 degrees C. Aviat. Space Environ. Med. 63, 107–113. 1546937

[B90] Mn S. (1992). Physiological consequences of hypohydration: exercise performance and thermoregulation. Med. Sci. Sports Exerc 24, 657–670. 10.1249/00005768-199206000-00008 1602938

[B91] Morrison S. Sleivert G. G. Cheung S. S. (2004). Passive hyperthermia reduces voluntary activation and isometric force production. Eur. J. Appl. Physiol. 91, 729–736. 10.1007/s00421-004-1063-z 15015001

[B92] Nassis G. P. Geladas N. D. (2002). Cardiac output decline in prolonged dynamic exercise is affected by the exercise mode. Pflugers Arch. 445, 398–404. 10.1007/s00424-002-0935-5 12466943

[B93] Nielsen B. Strange S. Christensen N. J. Warberg J. Saltin B. (1997). Acute and adaptive responses in humans to exercise in a warm, humid environment. Pflugers Arch. 434, 49–56. 10.1007/s004240050361 9094255

[B94] Numata U. Yanaoka T. Kurosaka S. Hasegawa H. (2023). Effects of ice slurry ingestion on body temperature and softball pitching performance in a hot environment: a randomized crossover trial. J. Physiol. Anthropol. 42, 12. 10.1186/s40101-023-00329-0 37386617 PMC10308695

[B95] Nybo L. Nielsen B. (2001). Middle cerebral artery blood velocity is reduced with hyperthermia during prolonged exercise in humans. J. Physiol. 534, 279–286. 10.1111/j.1469-7793.2001.t01-1-00279.x 11433008 PMC2278686

[B96] Otani H. Goto T. Goto H. Shirato M. (2017). Time-of-day effects of exposure to solar radiation on thermoregulation during outdoor exercise in the heat. Chronobiol Int. 34, 1224–1238. 10.1080/07420528.2017.1358735 28910548

[B97] Otani H. Kaya M. Tamaki A. Watson P. Maughan R. J. (2016). Effects of solar radiation on endurance exercise capacity in a hot environment. Eur. J. Appl. Physiol. 116, 769–779. 10.1007/s00421-016-3335-9 26842928

[B98] Pandolf K. B. (1998). Time course of heat acclimation and its decay. Int. J. Sports Med. 19 (Suppl. 2), S157–S160. 10.1055/s-2007-971985 9694426

[B99] Pandolf K. B. Cadarette B. S. Sawka M. N. Young A. J. Francesconi R. P. Gonzalez R. R. (1988). Thermoregulatory responses of middle-aged and young men during dry-heat acclimation. J. Appl. Physiol. 65, 65–71. 10.1152/jappl.1988.65.1.65 3403494

[B100] Périard J. D. Racinais S. Sawka M. N. (2015). Adaptations and mechanisms of human heat acclimation: applications for competitive athletes and sports. Scand. J. Med. Sci. Sports 25 (Suppl. 1), 20–38. 10.1111/sms.12408 25943654

[B101] Pryor R. R. Pryor J. L. Vandermark L. W. Adams E. L. Brodeur R. M. Armstrong L. E. (2019). Exacerbated heat strain during consecutive days of repeated exercise sessions in heat. J. Sci. Med. Sport 22, 1084–1089. 10.1016/j.jsams.2019.06.003 31235386

[B102] Racinais S. Alonso J. M. Coutts A. J. Flouris A. D. Girard O. González-Alonso J. (2015). Consensus recommendations on training and competing in the heat. Br. J. Sports Med. 49, 1164–1173. 10.1136/bjsports-2015-094915 26069301 PMC4602249

[B103] Racinais S. Gaoua N. Grantham J. (2008). Hyperthermia impairs short-term memory and peripheral motor drive transmission. J. Physiol. 586, 4751–4762. 10.1113/jphysiol.2008.157420 18703579 PMC2607529

[B104] Racinais S. Ihsan M. Taylor L. Cardinale M. Adami P. E. Alonso J. M. (2021). Hydration and cooling in elite athletes: relationship with performance, body mass loss and body temperatures during the Doha 2019 IAAF World Athletics Championships. Br. J. Sports Med. 55, 1335–1341. 10.1136/bjsports-2020-103613 33579722 PMC8606454

[B105] Racinais S. Oksa J. (2010). Temperature and neuromuscular function. Scand. J. Med. Sci. Sports 20 (Suppl. 3), 1–18. 10.1111/j.1600-0838.2010.01204.x 21029186

[B106] Ravanelli N. Coombs G. Imbeault P. Jay O. (2019). Thermoregulatory adaptations with progressive heat acclimation are predominantly evident in uncompensable, but not compensable, conditions. J. Appl. Physiology 127, 1095–1106. 10.1152/japplphysiol.00220.2019 31414952

[B107] Rhea M. R. (2004). Determining the magnitude of treatment effects in strength training research through the use of the effect size. J. Strength Cond. Res. 18, 918–920. 10.1519/14403.1 15574101

[B108] Rodríguez M. Á. Piedra J. V. Sánchez-Fernández M. Del Valle M. Crespo I. Olmedillas H. (2020). A matter of degrees: a systematic review of the ergogenic effect of pre-cooling in highly trained athletes. Int. J. Environ. Res. Public Health 17, 2952. 10.3390/ijerph17082952 32344616 PMC7215649

[B109] Rollo I. Randell R. K. Baker L. Leyes J. Y. Medina Leal D. Lizarraga A. (2021). Fluid balance, sweat Na+ losses, and carbohydrate intake of elite male soccer players in response to low and high training intensities in cool and hot environments. Nutrients 13, 401. 10.3390/nu13020401 33513989 PMC7912570

[B110] Rosinger A. Herrick K. (2016). Daily water intake among U.S. Men and women, 2009-2012. NCHS Data Brief, 1–8. 27139510

[B111] Saillant M. M. Charkoudian N. Salgado R. M. (2022). Individual variability in achievement of short-term heat acclimation during a fixed intensity protocol. J. Therm. Biol. 110, 103373. 10.1016/j.jtherbio.2022.103373 36462868

[B112] Sawka M. N. Burke L. M. Eichner E. R. Maughan R. J. Montain S. J. Stachenfeld N. S. (2007). American College of Sports Medicine position stand. Exercise and fluid replacement. Med. Sci. Sports Exerc 39, 377–390. 10.1249/mss.0b013e31802ca597 17277604

[B113] Sawka M. N. Young A. J. Francesconi R. P. Muza S. R. Pandolf K. B. (1985). Thermoregulatory and blood responses during exercise at graded hypohydration levels. J. Appl. Physiol. 59, 1394–1401. 10.1152/jappl.1985.59.5.1394 4066570

[B114] Sawka M. N. Young A. J. Latzka W. A. Neufer P. D. Quigley M. D. Pandolf K. B. (1992). Human tolerance to heat strain during exercise: influence of hydration. J. Appl. Physiol. 73, 368–375. 10.1152/jappl.1992.73.1.368 1506393

[B115] Sekiguchi Y. Adams W. M. Curtis R. M. Benjamin C. L. Casa D. J. (2019). Factors influencing hydration status during a National Collegiate Athletics Association division 1 soccer preseason. J. Sci. Med. Sport 22, 624–628. 10.1016/j.jsams.2018.12.005 30587437

[B116] Sekiguchi Y. Benjamin C. L. Giersch Gabrielle E. W. Belval Luke N. Stearns R. L. Casa D. J. (2021a). Practical implementation strategies for heat acclimatization and acclimation programming to optimize performance. Athl. Train. and Sports Health Care 0 13. 10.3928/19425864-20201002-01

[B117] Sekiguchi Y. Benjamin C. L. Manning C. N. Struder J. F. Armstrong L. E. Lee E. C. (2021b). Effects of heat acclimatization, heat acclimation, and intermittent exercise heat training on time-trial performance. Sports Health 14, 694–701. 10.1177/19417381211050643 34706597 PMC9460081

[B118] Shapiro Y. Moran D. Epstein Y. (1998). Acclimatization strategies--preparing for exercise in the heat. Int. J. Sports Med. 19 (Suppl. 2), S161–S163. 10.1055/s-2007-971986 9694427

[B119] Shirreffs S. M. Armstrong L. E. Cheuvront S. N. (2004). Fluid and electrolyte needs for preparation and recovery from training and competition. J. Sports Sci. 22, 57–63. 10.1080/0264041031000140572 14971433

[B120] Shirreffs S. M. Maughan R. J. (1997). Whole body sweat collection in humans: an improved method with preliminary data on electrolyte content. J. Appl. Physiology 82, 336–341. 10.1152/jappl.1997.82.1.336 9029235

[B121] Sonna L. A. Sawka M. N. Lilly C. M. (2007). Exertional heat illness and human gene expression. Prog. Brain Res. 162, 321–346. 10.1016/S0079-6123(06)62016-5 17645926

[B122] Stearns R. L. Casa D. J. Lopez R. M. McDermott B. P. Ganio M. S. Decher N. R. (2009). Influence of hydration status on pacing during trail running in the heat. J. Strength Cond. Res. 23, 2533–2541. 10.1519/JSC.0b013e3181b73c3f 19675477

[B123] Stephenson L. Kolka M. (1988). “Effect of gender, circadian period and sleep loss on thermal responses during exercise,” in Human performance physiology and environmental medicine at terrestrial extremes. Editors Pandolf K. B. Sawka M. N. Gonzalez R. R. (Indianapolis, IN: Benchmark Press), 267–304.

[B124] Stookey J. D. Kavouras S. Α. Suh H. Lang F. (2020). Underhydration is associated with obesity, chronic diseases, and death within 3 to 6 Years in the U.S. Population aged 51-70 years. Nutrients 12, 905. 10.3390/nu12040905 32224908 PMC7230456

[B125] Thomas M. M. Cheung S. S. Elder G. C. Sleivert G. G. (2006). Voluntary muscle activation is impaired by core temperature rather than local muscle temperature. J. Appl. Physiol. 100, 1361–1369. 10.1152/japplphysiol.00945.2005 16339343

[B126] Thornton S. N. (2014). Diabetes and hypertension, as well as obesity and Alzheimer’s disease, are linked to hypohydration-induced lower brain volume. Front. Aging Neurosci. 6, 279. 10.3389/fnagi.2014.00279 25352806 PMC4195368

[B127] Tyler C. J. Reeve T. Hodges G. J. Cheung S. S. (2016). The effects of heat adaptation on physiology, perception and exercise performance in the heat: a meta-analysis. Sports Med. 46, 1699–1724. 10.1007/s40279-016-0538-5 27106556

[B128] Wallis G. A. Rowlands D. S. Shaw C. Jentjens R. L. P. G. Jeukendrup A. E. (2005). Oxidation of combined ingestion of maltodextrins and fructose during exercise. Med. Sci. Sports Exerc 37, 426–432. 10.1249/01.mss.0000155399.23358.82 15741841

[B129] Ward P. Coutts A. J. Pruna R. McCall A. (2018). Putting the “I” back in team. Int. J. Sports Physiol. Perform. 13, 1107–1111. 10.1123/ijspp.2018-0154 29952669

[B130] Wegmann M. Faude O. Poppendieck W. Hecksteden A. Fröhlich M. Meyer T. (2012). Pre-cooling and sports performance: a meta-analytical review. Sports Med. 42, 545–564. 10.2165/11630550-000000000-00000 22642829

[B131] Weller A. S. Linnane D. M. Jonkman A. G. Daanen H. A. M. (2007). Quantification of the decay and re-induction of heat acclimation in dry-heat following 12 and 26 days without exposure to heat stress. Eur. J. Appl. Physiol. 102, 57–66. 10.1007/s00421-007-0563-z 17891541

[B132] Wickham K. A. Wallace P. J. Cheung S. S. (2021). Sex differences in the physiological adaptations to heat acclimation: a state-of-the-art review. Eur. J. Appl. Physiol. 121, 353–367. 10.1007/s00421-020-04550-y 33205218

[B133] Yeargin S. W. Casa D. J. McClung J. M. Knight J. C. Healey J. C. Goss P. J. (2006). Body cooling between two bouts of exercise in the heat enhances subsequent performance. J. Strength Cond. Res. 20, 383–389. 10.1519/R-18075.1 16686568

